# Experiments on physical ablation of long bone using microwave ablation; defining optimal settings using ex- and in-vivo experiments

**DOI:** 10.1371/journal.pone.0284027

**Published:** 2023-04-07

**Authors:** Hendricus Nijland, Jinwen Zhu, Thomas Kwee, Dingjun Hao, Paul Jutte

**Affiliations:** 1 Department of Orthopaedic Surgery, University Medical Center Groningen, Groningen, The Netherlands; 2 Department of Orthopaedic Surgery, Xi’an Honghui hospital, Xi’an, China; 3 Department of Radiology, University Medical Center Groningen, Groningen, The Netherlands; Jeonbuk National University, REPUBLIC OF KOREA

## Abstract

**Background:**

Improved survival of cancer patients leads to more skeletal metastatic lesions that need local therapies for tumor control and pain relief. Not all tumors are radiosensitive and alternative therapies are direly needed. Microwave ablation (MWA) is a technique for minimally invasive local tumor control by physical ablation. In soft tissue local temperature ablation is more common, but studies on bone tissue are limited. To ensure safe and effective treatment, studies on local tumor ablation in bone are needed.

**Method:**

Microwave ablation was performed on sheep bone, for both in- and ex-vivo settings. Both a slow-cooking MWA protocol (gradually increasing wattage in the first two minutes of ablation) and a fast-cooking protocol (no warm-up period) were used. Heat distribution through the bone during ablation was determined by measuring temperature at 10- and 15mm from the ablation probe (= needle). Ablation size after procedure was measured using nitro-BT staining.

**Results:**

In-vivo ablations led to up to six times larger halos than ex-vivo with the same settings. Within both ex- and in-vivo experiments, no differences in halo size or temperature were found for different wattage levels (65W vs 80W). Compared to a fast cooking protocol, a two-minute slow cooking protocol led to increased temperatures and larger halos. Temperatures at 10- and 15mm distance from the needle no longer increased after six minutes. Halo sizes kept increasing over time without an evident plateau.

**Conclusion:**

Microwave ablation is technically effective for creating cell death in (sheep) long bone. It is recommended to start ablations with a slow-cooking period, gradually increasing the surrounding tissue temperature in two minutes from 40 to 90°C. Ex-vivo results cannot simply be translated to in-vivo.

## Introduction

Improved survival of cancer patients leads to more skeletal metastatic lesions that are amenable to local therapies for tumor control and pain relief [1]. Not all tumors are radiosensitive and alternative therapies are direly needed. Microwave ablation (MWA) is a technique for minimally invasive tumor treatment. It is frequently used for local control in soft tissue malignancies like liver-, kidney- and lung cancer lesions [2,3]. Heat distribution depends on power (watts), ablation time (minutes) and bone properties. Higher power and longer time likely lead to larger ablation areas (halo). Literature on relations between time and energy have so far only been published for radiofrequency ablation [4]. Therefore, optimal settings for individual lesions are unknown. Safe and effective procedures cannot be done without those studies.

**Fig 1:**
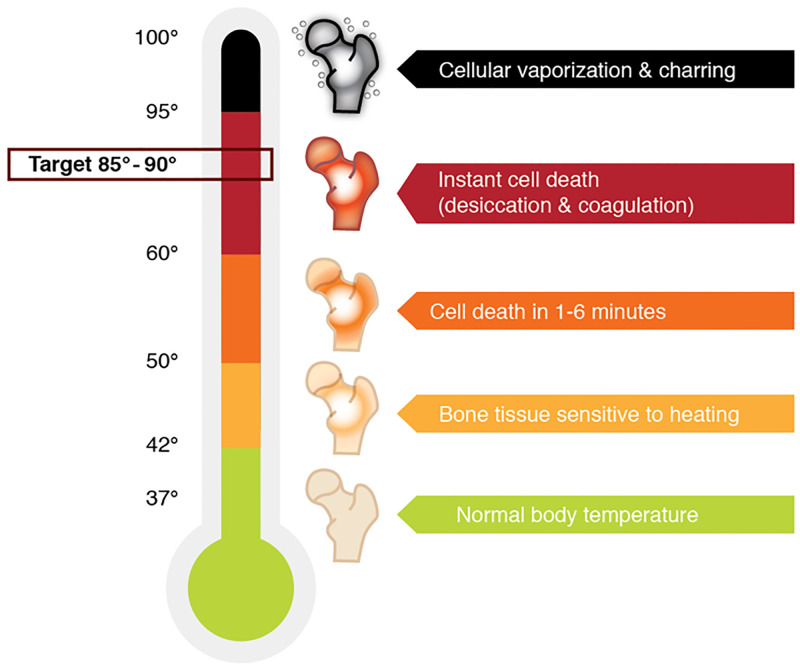
Tissue reaction to heat [15]. Ideal temperature for ablation is 60–95°C since instant cell death is the aim. Below 60°C cells may recover. Above 100°C carbonization occurs, limiting distribution of heat.

At temperatures over 60°C instant cell death occurs by coagulative necrosis. Between 50–60°C it takes 1–6 minutes to reach complete cell death [5–7]. Therefore, to ensure complete ablation, temperature at the outer edge of the tumor should reach over 60°C. In the surrounding areas heat-induced stress leads to apoptosis by disturbance in mitochondrial pathways and caspase activation. Furthermore reactive oxygen species (ROS) create oxidative damage and disturb mitochondrial functioning thereby altering the rate of apoptosis [8–10]. The cell is protected against heat-induced apoptosis by activation of heat shock proteins [11]. They inhibit caspase activation and stabilize proteins, thereby confirming correct folding (chaperone function). In clinical procedures temperature is aimed at 90°C to ensure temperature over 60*°*C in the complete tumor. It is expected that temperature decreases during conduction through the tissue as a result of (micro)vascularity. After ablation with temperatures under 60*°*C tissue has the capability to recover, leading to possible tumor recurrence. At temperatures over 100°C vaporization of water (leading to desiccation of the cell) and carbonization occur. These factors potentially limit heat conduction [12–14]. In Fig 1, a model is shown depicting the tissue reaction to increasing temperatures [15].

Literature on safety and efficacy of local temperature ablation in bone tissue is still limited. A wide range of wattage levels is used for clinical MWA. Given the fact MWA leads to larger ablation zones compared to alternatives like radiofrequency ablation, clinical use might result in excessive margin or incomplete ablation. An excessive margin could lead to damage to surrounding structures, incomplete ablation to lack of local control [16]. To ensure safe and effective ablation it is essential to develop a reliable prediction of the ablation area for different settings.

In this study we compared different settings to study the effects of wattage level and ablation time on halo size.

## Method

### Ablation procedure

All ablations were performed with a 2.45GHz Kang-You 2000 microwave ablation generator (Kang-You Medical, Nanjing, China). The needle had an active part of three cm long and a diameter of 17 gauge. Internal cooling of the needle was performed, preventing temperatures over 45°C in the non-active part. Needles were cleaned with alcohol between procedures and replaced when the protective insulation layer started to show damage. An ex-vivo study was performed to determine heat distribution and halo size for a wide range of settings. Subsequently the most promising settings were transferred into an in-vivo study.

### Ex-vivo

Fresh sheep femur (from sheep aged 1–1.5 years with an average weight of 50kg) were collected from the slaughterhouse. These bones were chosen given the similarity to human bone anatomy. The lower extremity is the most common location for metastases and tumors in human bone [17]. Ablation was performed within 6–12 hours after sacrifice to ensure bone quality was as close to physiological and vital bone as possible. First, the bones were put into a reservoir containing water with an aimed temperature of 37°C (range 37–40°C) for 20–30 minutes to mimic body circumstances. Before starting ablation, inner bone temperature was controlled.

To prevent carbonization as a result of over-rapid temperature increase around the needle tip (which is thought to limit heat distribution), a protocol for slow-cooking was developed. According to this protocol wattage was slowly increased towards the aimed setting: 10W for one minute, followed by one minute at 20W. These two minutes were included in the time intervals as mentioned for the slow-cooking ablations (see next paragraph). Furthermore, a fast-cooking protocol was developed. In this protocol ablation was directly started at the aimed maximum wattage. Results of both protocols were compared.

#### Heat distribution

**Fig 2:**
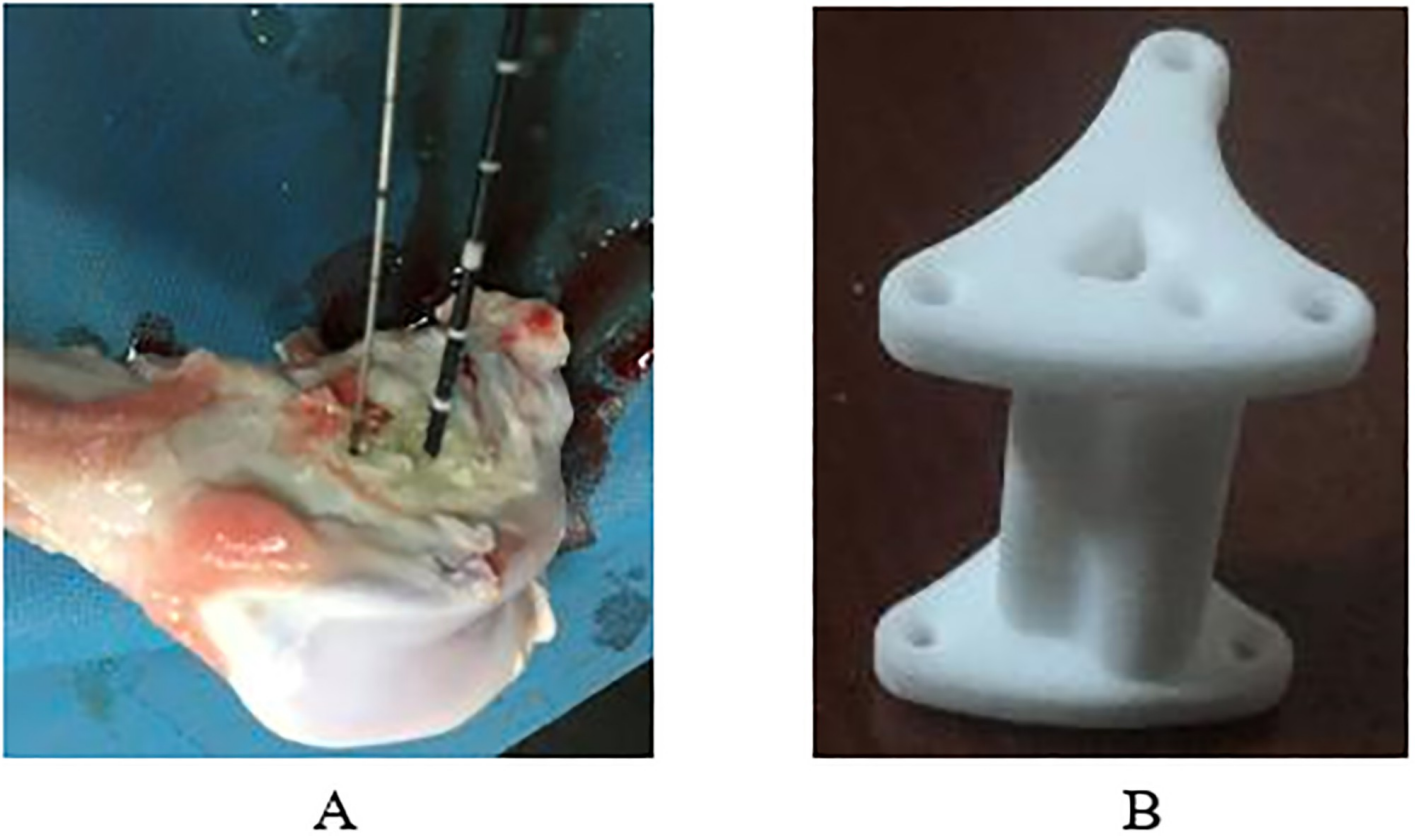
**A.** Ablation in a sheep femur metaphysis with temperature probe at 10mm. **B.** The 3D-printed navigation tool.

The tract for the ablation needle was drilled in the proximal metaphysis of sheep femur at a depth of 30mm. Through this tract the ablation needle was brought up. Temperature was measured by a Kang-You temperature probe (21 gauge). Tracts for the temperature probe were drilled 5, 10 or 15mm lower in the same direction as the ablation needle (see Fig 2A). Only one distance was measured per ablation session to prevent heat leakage. During ablation, temperature was live recorded. A 3D-printed navigation tool (see Fig 2B) was designed to standardize distance and needle direction, thereby minimizing variation between measurements. The tool was made from PA12 nylon and had holes with a diameter of two mm at 5-, 10- and 15mm from a central tunnel.

A total of ten settings was used, with wattages of 65W (6 and 12 minutes fast-cooking and 6, 8, 10, 12 minutes slow cooking) and 80W (6, 8, 10, 12 minutes slow-cooking). The aim of this was to determine the contribution of each parameter or setting on halo size. For each setting 10 measurements were performed.

**Fig 3:**
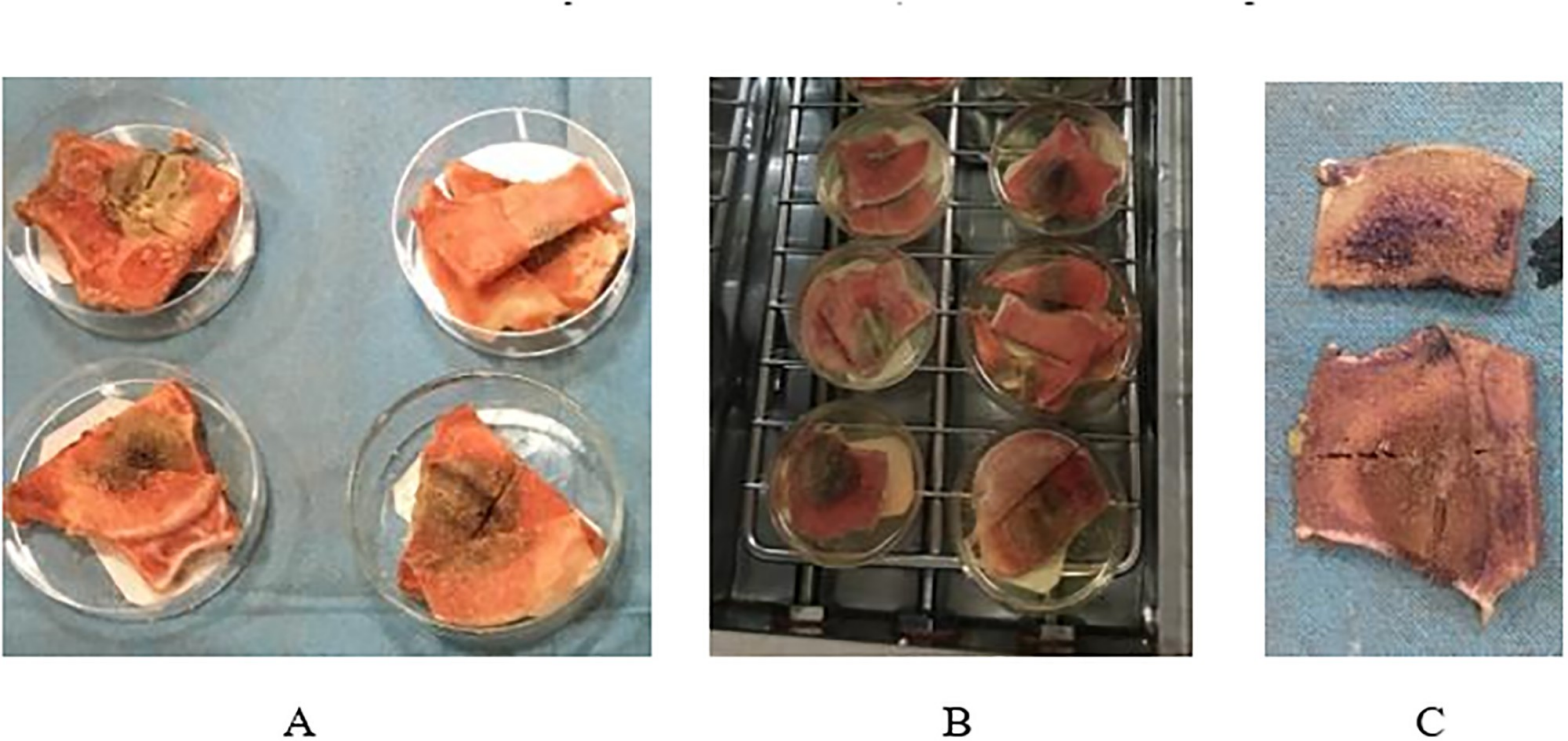
Staining of sheep proximal femur metaphysis samples after ablation. **A.** Samples before staining. **B.** Samples in the incubator. **C.** Sample after staining in Nitro-BT solution.

Between two and eight hours after ablation the bones were cut in the coronal plane at the insertion point of the needle using an automatic saw with a 1-mm thick blade. Subsequently the coupes were stained in a Nitro-BT solution (nitro blue tetrazolium chloride) and incubated in a 37°C incubator for three hours (see Fig 3B). This solution was made according to Nachlas et al. using eight parts of H_2_0, one part phosphate buffer solution and one part Nitro-BT [18]. As a result of staining, healthy tissue turns purple (see Fig 3C). Dimensions before and after staining were measured and compared. Measurements were corrected with 1mm to correct for the bone loss by the saw blade (1mm loss was objectivated in our trials).

### In-vivo

In-vivo MWA was performed in 12 sheep femurs, using the same protocol as the ex-vivo experiments. For four sheep ablation was performed with 65W for six minutes, for four sheep 65W for 12 minutes and for four sheep 80W for six minutes. Heat distribution was measured at 10- and 15mm for 65W ablations (one distance per ablation) and at 10mm for 80W ablations (not at 15mm based on ex-vivo results and to limit the number of sheep needed). Before starting the procedure, the sheep were sedated by a veterinarian with an intramuscular injection of 5 ml Shutai (1: 1 combination of Tiletamine 50mg/ml & Zolazepam hydrochloride 50mg/ml) (Virbac, France) and subsequently intubated. During the procedure inhaled isoflurane (1–5%) was used for general anesthesia and a maintenance dose up to 2.5ml Shutai was given in case deemed necessary by the veterinarian. Sheep were sacrificed one week after the procedure, bones were cut and halo size was determined after staining (with the same procedure as ex-vivo). The in-vivo study was approved by the medical ethical committee of the Xi’an HongHui hospital.

### Data analysis

Technical issues like sudden temperature drops, temperature differences more than two standard deviations (SDs) from the average value (for at least three time intervals) and high impedance (dessicated tissue in a few ex-vivo bones) warranted exclusion from the analysis.

Data were analyzed using SPSS v25 (IBM, Armonk, United States). Values for temperature and halo dimensions were noted as mean (±SD). Halo volume was calculated according to the formula: 4/3*pi*(1/2*height)*(1/2*width)*(1/2*depth) = 1/6*pi*height*width*depth. Data were examined using non-parametric tests due to the relatively small group size. Differences between fast- and slow-cooking, 65W and 80W, and in- and ex-vivo were tested using a mixed repeated measures ANOVA design. Sphericity and equality of differences were controlled. Data were tested for time up to six minutes (after six minutes temperature remained stable and variance at these intervals is very limited). For all tests a p-value of < .05 was considered significant.

## Results

### Ex-vivo heat distribution

**Fig 4:**
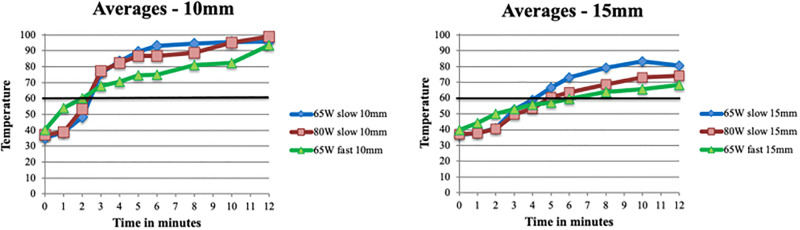
Average temperatures at different distances from the needle for MWA in ex-vivo sheep femur. **A.** Averages at 10mm for 65W slow- and fast cooking and 80W slow-cooking. **B.** Averages at 15mm for 65W slow- and fast cooking and 80W slow-cooking. Black lines depict the aimed temperature of 60°C.

For the slow-cooking samples, temperature remained below 60°C during the two-minute slow-cooking period. For both 65W and 80W temperature exceeded 60°C within three minutes at 10mm and within five minutes at 15mm. There was no significant difference in temperature over time between 65W and 80W (at 10mm F(5,55) = 1.43, p = .228, at 15mm F(5,60) = 1.68, p = .152). For the fast-cooking samples, initial temperature increase was fast with temperature reaching up to 60°C within two minutes at 10mm from the needle. At 15mm temperature increase was similar to the slow-cooking samples. After the initial fast increase, temperatures increased more gradually compared to the slow-cooking samples. From five minutes onwards temperature was significantly lower at both 10mm (F(5,55) = 35.19, p < .01) and 15mm (F(5,65) = 7.23, p < .01). The values are depicted in Table 1 and Fig 4A and 4B).

**Table 1:** Halo volume vs time for different settings for microwave ablation. Blue = 65W slow-cooking, red = 80W slow-cooking, green = 65W fast-cooking.

*Setting*	*Height (mm)*	*Width (mm)*	*Depth (mm)*	*Volume (cm* ^*3*^ *)*
*65W*, *6 min*, *fast*	14.7 (±0.58)	20.3 (±1.15)	14.3 (±1.53)	2.23 (±0.36)
*65W*, *12 min*, *fast*	19.8 (±3.13)	19.0 (±1.10)	18.7 (±2.42)	3.68 (±1.10)
*65W*, *6 min*, *slow*	14.7 (±2.08)	20.3 (±3.06)	16.0 (±1.00)	2.50 (±0.36)
*65W*, *8 min*, *slow*	16.0 (±1.84)	20.8 (±1.83)	17.8 (±2.68)	3.10 (±0.83)
*65W*, *10 min*, *slow*	16.5 (±3.33)	21.4 (±1.80)	19.5 (±2.38)	3.61 (±0.95)
*65W*, *12 min*, *slow*	17.6 (±3.57)	21.4(±1.77)	20.2(±2.07)	3.98 (±1.23)
*80W*, *6 min*, *slow*	13.0 (±1.87)	20.2 (±1.92)	19.6 (±1.14)	2.69 (±0.66)
*80W*, *8 min*, *slow*	14.6 (±2.64)	20.8 (±2.31)	18.5 (±4.41)	2.94 (±0.70)
*80W*, *10 min*, *slow*	14.9 (±2.97)	20.3 (±1.04)	18.3 (±3.01)	2.90 (±0.90)
*80W*, *12 min*, *slow*	16.7 (±2.26)	21.6 (±1.75)	19.5 (±2.45)	3.68 (±0.75)

### Ex-vivo halo size

Halo volume gradually increased over time. The major part of the halo was formed within the first six minutes. Halo size continued to increase up to the 12-minute measurement but was not significantly larger at twelve minutes compared to six minutes for the slow cooking samples (65W p = .067, 80W p = .421). For the fast cooking samples halo size after 12 minutes was significantly larger (p = .024). This is contrary to the heat distribution data where almost no further increase was seen in the period after six minutes. There was no difference in halo size between 65W and 80W (p = .890) or between fast- and slow-cooking (p = .861). Values for volume are depicted in Table 1.

### In-vivo heat distribution

**Fig 5:**
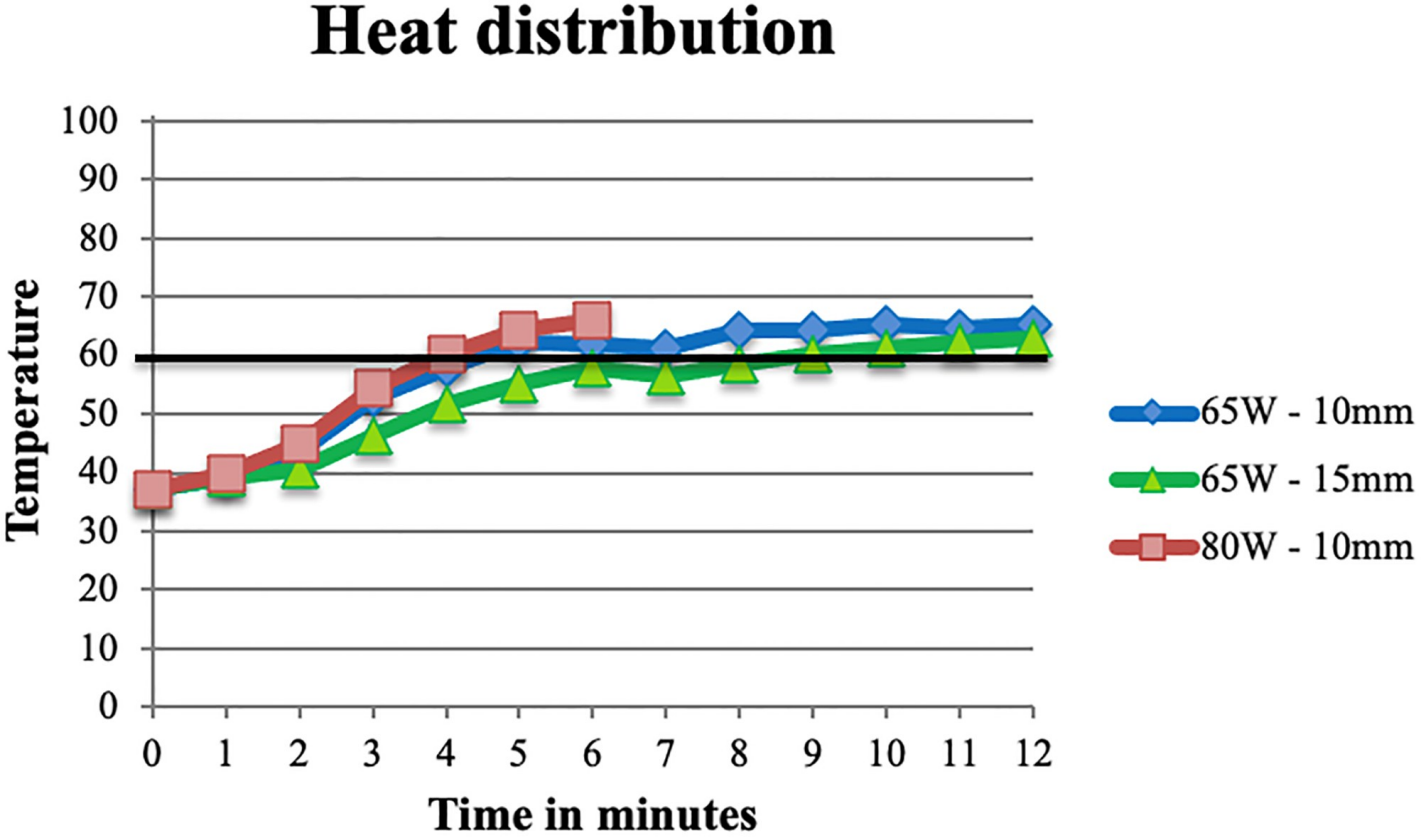
Heat distribution for in-vivo MWA in sheep femur. Blue = 65W—10mm, green 65W—15mm, red = 80W—10mm. Black line depicts the aimed temperature of 60°C.

For in-vivo ablations, heat distribution was significantly smaller compared to ex-vivo (at 10mm F(5,65) = 17.69, p < .01, at 15mm F(5,65) = 6.58, p < .01). At 10mm temperature reached over 60°C after four to five minutes (for 80W and 65W respectively), at 15mm (with 65W) this occurred after nine minutes. Temperature remained below the ‘safe’ 60°C for a longer time than in ex-vivo. Values are depicted in Fig 5.

### In-vivo halo size

**Fig 6:**
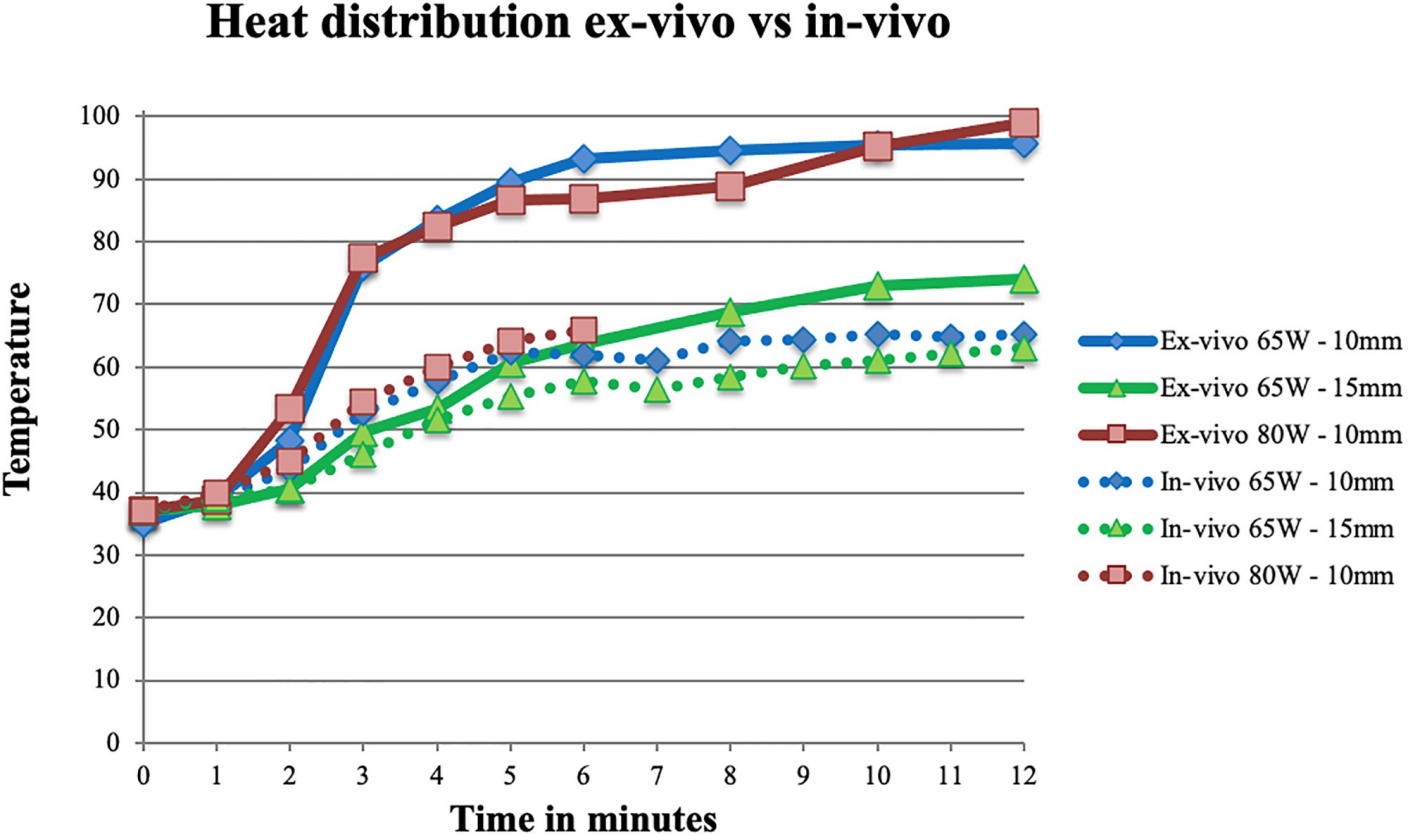
Heat distribution ex-vivo vs in-vivo. In-vivo temperatures are significantly lower.

Halo size in-vivo was significantly larger than ex-vivo (p < .01). Halo size did not significantly increase between six and twelve minutes ablation (p = .857). There was no significant difference in halo size between 65W and 80W either (p = .057). Table 2 depicts the mean dimensions for in-vivo ablation. In Fig 6 the heat distribution ex-vivo is compared to in-vivo.

**Table 2:** Halo size in vivo.

*Setting*	*Height (mm)*	*Width (mm)*	*Depth (mm)*	*Volume (cm* ^*3*^ *)*
*65W*, *6 min*	35.3 (±6,11)	33 (±8,37)	30 (±9,20)	23.0 (±5.42)
*65W*, *12 min*	34.8 (±11,8)	39.0 (±2,16)	40.8 (±6,55)	28.9 (±11.0)
*80W*, *6 min*	29.3 (±0,96)	30.0 (±4,08)	31.0 (±5,89)	14.4 (±4.10)

## Discussion

This study is the first to examine the effect of different settings for microwave ablation in long bone in an experimental setting. Since both ex-vivo and in-vivo experiments were performed, results are likely transferrable to the clinical setting. Results prove microwave ablation is technically effective for creating cell death in (sheep) long bone in a minimally invasive manner. Ablation with 65W is sufficient, the major portion of cell death is formed in the first six minutes of ablation, slow-cooking is better than fast-cooking and ex-vivo halo size is smaller than in-vivo halo size. This data indicates ‘aggressive’ ablation with high wattages and longer time intervals does not lead to better results. To reduce risk of complications ablation settings must be determined with caution.

Assumptions regarding the relationship between cell death and temperature as postulated in literature (see Fig 1) do not match with the current (ex-vivo) data. According to literature, cell death occurs instantly at temperatures over 60°C or after 1–6 minutes at 50–60°C (5–7, 18). In the present study, temperatures measured at 10mm from the needle exceeded 60° C within three minutes for ex-vivo ablations. This indicates a 20mm diameter halo with temperature over 60°C, when assuming equal distribution of heat through the bone. The ablation halo only reached a diameter of 20mm after 10–12 minutes in the ex-vivo experiments. Therefore, there seems to be a delay between temperature over 60°C and corresponding cell death. This is in line with the fact that temperature at 10- and 15mm from the needle does not further increase after six minutes, whereas halo size gradually does. A probable explanation for this is slow growing heat flow due to conduction of the tissue. This becomes slower as tissue loses its water content. However, halo size after 12 minutes was not significantly larger than after six minutes (p = .857). In-vivo halo size corresponded better with the expected halo size based on assumptions of cell death as described in Fig 1 than ex-vivo (cell death at temperatures over 60°C). Temperatures at 15mm were close to 60°C after six minutes. Halo dimensions around 30mm for height, width and depth correspond to this (when assuming equal distribution of heat through the bone).

### Heat distribution

Temperatures at 10- and 15mm from the needle were higher ex-vivo than in vivo. This is most likely due to in-vivo heat sink, for instance by (micro)vascularity in and around the bone. In the study of Ji et al. they measured temperature at 5, 10, 15 and 20mm (parallel array of probes) from the needle tip during a 36-minute microwave ablation in dog femur (amount of Watt not described) [19]. They found temperature to increase rapidly in the first six minutes leading to temperatures of 86.4°C at 5mm, 74.0°C at 10mm and 54.1°C at 15mm after six minutes. At 20mm, temperature only reached 42.0°C. In the next 30 minutes temperature only increased with another 5–10% at all distances. A limitation of their study is that they used a parallel array of probes. This potentially creates a large artificial heat sink (dependent of thermal couple properties) because the metal of the antenna will conduct heat more easily than the surrounding bone, leading to a significant amount of heat loss. Furthermore, they only used one ablation setting and a small number of cases.

### Halo size

Interestingly, halo size was larger in the in-vivo samples than ex-vivo, regardless of the lower temperatures at 10- and 15mm from the needle. A possible explanation for the difference between in- and ex-vivo halo size is the time between ablation and halo measurement. In the in-vivo experiment the animals were sacrificed after one week and subsequently halo size was measured whereas ex-vivo measurement was done within hours after the ablation. The cells that were identified dead in the in-vivo experiment could be the effect of both necrosis as well as apoptosis, whereas in the ex-vivo experiment only the effect of necrosis can be seen (since analysis was done shortly after ablation). There might also be an effect of late-apoptosis in the in-vivo experiments, for instance because of ROS activity. However, it is not likely that differences as large as found in our data only originate from follow-up time after ablation [20,21].

### Settings

An increase in wattage level from 65W to 80W did not lead to larger halos or higher temperatures at 10- and 15mm from the needle. Therefore, a wattage of 65W is considered sufficient. Further increase of the wattage only increases risk of complications. Potentially a similar outcome could be found for even lower wattage levels. This could be examined in further research. Between fast- and slow cooking significant differences in heat distribution were seen. In the fast-cooking group temperature at 10- and 15mm is higher during the first two minutes of ablation compared to slow-cooking. However, from five minutes onwards the temperature of the slow-cooking group is significantly higher. This effect is hypothesized to be the result of carbonization around the needle as a result of over-rapid heating in the fast-cooking samples. This carbonized tissue can (partially) block the distribution of heat through the bone. Therefore, we recommend to use a two-minute slow-cooking period at every ablation to ensure gradual heating of the tissue. Since halo size mainly increased in the first six minutes, longer ablations than six minutes are not encouraged.

This study had some limitations. First, positioning of the drill was done by hand and without image-guidance. Therefore, small differences between the measurements cannot be ruled out. For all ex-vivo experiments at least seven samples were included to ensure a reliable outcome. Distance between needle and temperature probe was standardized using a navigation tool, thereby also ensuring same direction and limiting variation between samples as a result of human error. This tool was also used for the in-vivo experiments. Second, for the in vivo experiments only four animals were used per setting and there was substantial variance in halo size between the animals. For the in-vivo study animals were sacrificed after one week and subsequently analyzed whereas ex-vivo analysis was performed on the day of ablation. Finally, experiments were performed in healthy bone without tumor tissue.

## Conclusion

Microwave ablation is technically effective for creating cell death in (sheep) long bone. It is recommended to start ablations with a slow-cooking period, gradually increasing the surrounding tissue temperature in two minutes from 40 to 90°C. Ex-vivo results cannot simply be translated to in-vivo, given in-vivo halo volume was up to six times larger than ex-vivo. The technique still faces issues like high variability and therefore limited predictability, before it can become mainstream treatment. Future experiments and development should focus on ways to reduce this.

## Supporting information

S1 Data set (XLSX)
